# Wnt/β-catenin signaling stimulates the expression and synaptic clustering of the autism-associated Neuroligin 3 gene

**DOI:** 10.1038/s41398-018-0093-y

**Published:** 2018-03-05

**Authors:** Matías A. Medina, Víctor M. Andrade, Mario O. Caracci, Miguel E. Avila, Daniela A. Verdugo, Macarena F. Vargas, Giorgia D. Ugarte, Ariel E. Reyes, Carlos Opazo, Giancarlo V. De Ferrari

**Affiliations:** 1 Center for Biomedical Research, Faculty of Biological Sciences and Faculty of Medicine, Universidad Andres Bello, Santiago, Chile; 2 Department of Biological Sciences, Faculty of Biological Sciences, Universidad Andres Bello, Santiago, Chile; 3 Oxidation Biology Laboratory, The Florey Institute of Neuroscience and Mental Health, The University of Melbourne Parkville, Melbourne, Victoria Australia; 4 Present Address: Institute of Natural Sciences, Universidad de las Américas, Santiago, Chile

## Abstract

Synaptic abnormalities have been described in individuals with autism spectrum disorders (ASD). The cell-adhesion molecule Neuroligin-3 (Nlgn3) has an essential role in the function and maturation of synapses and NLGN3 ASD-associated mutations disrupt hippocampal and cortical function. Here we show that Wnt/β-catenin signaling increases Nlgn3 mRNA and protein levels in HT22 mouse hippocampal cells and primary cultures of rat hippocampal neurons. We characterized the activity of mouse and rat Nlgn3 promoter constructs containing conserved putative T-cell factor/lymphoid enhancing factor (TCF/LEF)-binding elements (TBE) and found that their activity is significantly augmented in Wnt/β-catenin cell reporter assays. Chromatin immunoprecipitation (ChIP) assays and site-directed mutagenesis experiments revealed that endogenous β-catenin binds to novel TBE consensus sequences in the Nlgn3 promoter. Moreover, activation of the signaling cascade increased Nlgn3 clustering and co- localization with the scaffold PSD-95 protein in dendritic processes of primary neurons. Our results directly link Wnt/β-catenin signaling to the transcription of the Nlgn3 gene and support a functional role for the signaling pathway in the dysregulation of excitatory/inhibitory neuronal activity, as is observed in animal models of ASD.

## Introduction

Autism spectrum disorders (ASD) are highly heterogeneous, pervasive neurodevelopmental disorders characterized by persistent impairments in reciprocal social communication and repetitive patterns of behaviors.^1^ Although its etiology is unknown, ASD prevalence appears to be increasing.^2–5^ Family and twin studies have established ASD as highly inheritable diseases with a 90% phenotypic concordance among monozygotic twins.^6^ ASD mutations range from either common or *de novo* single nucleotide to copy number variants, to large-scale DNA deletions, duplications or translocations.^7,8^ According to SFARI Gene^9^ there are more than 800 genes implicated in ASD, with at least 50 high-ranking candidate risk genes, including the Neuroligin-3 gene (*NLGN3*: Gene ID: 54413, ChrXq13.1), which is strongly enriched with variants that affect ASD risk.^10–13^

Neuroligins (Nlgns) are postsynaptic cell-adhesion molecules that act as ligands for presynaptic Neurexins (Nrxns).^14–16^ Five different NLGN genes have been described in humans (NLGN1-3/4X/4Y), and since the first description of NLGN3 and NLGN4 as candidate genes for ASD^10^ all other NLGN genes have been associated with the disorder.^17–19^ Moreover, genes coding for Nlgn-interacting partners in the postsynaptic density, such as some NRXN^20–22^ and SHANK family members, have also been implicated with ASD,^23–25^ supporting an essential role for Nlgn complexes in the onset or development of ASD. At the functional level, Nlgns enhance the formation and maturation of specific types of synapses. For instance, while Nlgn1 is found preferentially in excitatory and Nlgn2 in inhibitory and cholinergic synapses,^26,27^ Nlgn3 localizes at both excitatory and inhibitory synapses.^28–32^ Nlgn4X has been observed in inhibitory glycinergic synapses.^33^ Currently, mice expressing Nlgn3 mutated forms display autism-like behaviors and are highly sought as experimental models for the study of ASD pathology.^34,35^

We predicted earlier that sustained gain-of-function of Wnt/β-catenin signaling in the developing brain could be responsible for the onset/development of ASD and that this effect involves the additive effect of genetic variants within components and/or genes whose products modulate its functional activity.^36^ This hypothesis has received considerable attention recently,^37–40^ mainly since it has been observed that 39% of the more disruptive *de novo* mutations in ASD family trios were found within interconnected networks containing chromatin remodeling, synaptic, and Wnt/β-catenin signaling genes.^41,42^ Likewise, integration of RNA-seq expression profiles during brain development with protein–protein interaction networks have identified highly modules enriched of connected Wnt signaling genes associated with ASD.^43^ At the genetic level, several Wnt/β-catenin components have been associated with ASD, including the canonical Wnt2 ligand,^44^ Wnt/β-catenin target genes Engrailed 2 (*EN2*)^45^ and the hepatocyte growth factor receptor (*MET*),^46–48^ and cadherins encoding genes such as *CDH5*, *CDH8*, *CDH9*, *CDH10*, *CDH13*, *CDH15*, *PCDH10*, *PCDH19,* and *PCDHb4*,^49^ some of which interact with β-catenin in cell-cell adhesion complexes. More recently, the chromo-helicase domain protein 8 (*CHD8*),^41,50,51^ which inhibits β-catenin through direct binding,^52^ and *DYRK1A* that modulates Wnt/β-catenin signaling through interaction with the p120 catenin,^53^ have been found to be associated with ASD. Interestingly, *CHD8* and *DYRK1A* harbor recurrent disruptive mutations and are highly correlated with head size abnormalities,^51^ a feature commonly observed in ASD. Moreover, rare *de novo* genetic variants in the β-catenin (*CTNNB1*) gene itself have been implicated in severe intellectual disability.^54^

Further support for a role of Wnt/β-catenin signaling in ASD comes from pharmacological studies, research in ASD comorbidities such as Tuberous sclerosis or in the recognition that an abnormal immune response plays an important role in the onset or development of the disorder.^55–57^ First, in utero exposure to teratogens such as valproic acid (valproate; VPA) causes a higher incidence of ASD in the offspring^58,59^ and VPA is known to increase cytosolic and nuclear β-catenin levels and activate Wnt/β-catenin-dependent gene expression, by a complex mechanism involving inhibition of histone deacetylase (HDAC) and glycogen synthase kinase-3α/β (GSK3α/β) activities.^60,61^ Second, in Tuberous sclerosis the tumor suppressor complex formed by hamartin (*TSC1* in Chr 9) and tuberin (*TSC2* in Chr 16) interact with the β-catenin degradation complex and thus modulates the action of Wnt/β-catenin signaling.^62,63^ Finally, several inflammatory cytokines, including interleukin 6 (IL-6), tumor necrosis factor-α (TNF-α), transforming growth factor-β1 (TGF-β1) and interferon γ (IFN-γ), are elevated in peripheral blood cells, serum, plasma, cerebrospinal fluid, or in the brains of ASD children.^64–72^ In this context, recent evidence indicates that Wnt/β-catenin and non-canonical Wnt signaling have both pro- and anti-inflammatory activity^73^ and that the signaling cascade is involved in inflammation-driven brain damage and inflammation-directed brain repair.^74^ Wnt3a selectively increases the expression of proinflammatory immune response genes in microglia and enhances the release of *de novo* IL-6, IL-12, and TNF-α.^75^ Nevertheless, the transcriptional program elicited by Wnt/β-catenin signaling in different types of brain cells has received little attention.^76–79^

Therefore, considering that Wnt/β-catenin signaling has an essential role in ASD affected regions such as the frontal cortex and the hippocampal formation,^80,81^ that others and we have observed that Wnt/β-catenin signaling enhances excitatory neurotransmission in hippocampal neurons,^82–85^ and that the transcriptional program controlling Nlgn3 expression and function is currently unknown, here we investigated the functional effects of the signaling cascade on Nlgn3 expression and synaptic function in hippocampal neurons.

## Materials and methods

### Primary cultures of rat hippocampal neurons

Primary hippocampal neurons were obtained from E18 Sprague-Dawley rat embryos (randomly selected; male/female in equal proportion) as described.^84,86^ Cells were maintained for 14 days in vitro (DIV) on culture plates (3.5 × 10^5^ cells/plate) coated with poly-l-lysine (Sigma-Aldrich, St. Louis, MO, USA). Primary neurons were grown on Neurobasal medium supplemented with B27 (Gibco BRL, Thermo Fisher Scientific, Waltham, MA, USA) and 50% media was replaced every 3 days. Cells were kept at 37 °C in 5% CO_2_ incubator and saturated humidity. The study protocol with rat embryos was approved by the Bioethical Committee of Universidad Andres Bello, Chile (026/2013) and was conducted in accordance with the Ethical Guidelines for Treatment of Laboratory Animals of the National Commission on Science and Technology (CONICYT- Chile).

### Cell lines

HT22 mouse hippocampal cells^87–90^ (a gift from Dr. Randall T. Moon, University of Washington, WA, USA), HEK293T, Wnt3a-L-cells mouse fibroblasts and control-L-cells (CRL-3216, CRL-2647, and CRL-2648, respectively; ATTC, Rockville, MD, USA), were maintained in DMEM supplemented with 10% FBS and 1% penicillin/streptomycin (Gibco BRL, Thermo Fisher Scientific), 1% P/S (Invitrogen, Thermo Fisher Scientific) and kept at 37 °C in 5% CO_2_ incubator with saturated humidity.

### Wnt3a purification

Wnt3a is a specific Wnt/β-catenin signaling agonist that can be efficiently recovered from conditioned medium from Wnt3a-secreting L-cells (Wnt3a-CM). Wnt3a purification was carried out, as previously described.^84,91,92^ The presence of the Wnt3a protein was detected with an anti- Wnt3a antibody (R & D Systems, Minneapolis, MN, USA). Purity was analyzed by SDS–PAGE (8%), stained with Coomassie Blue G250, and analyzed through densitometry by using software ImageJ.^93^

### Semi- and quantitative-PCR (q-PCR) analysis

Primary cultures of hippocampal neurons or HT22 cells were seeded in 6 well culture plates (5 × 10^5^ cells per ml) and stimulated with 200–400 ng/ml of purified Wnt3a protein or with 10–20 mM LiCl (Sigma-Aldrich) and collected for mRNA or protein analyses, as described.^94,95^

Briefly, total RNA was extracted in RNase free conditions using TRIzol reagent (Thermo Fisher Scientific) and 2 µg of RNA was reverse transcribed with Affinity Script QPCR cDNA synthesis kit (Agilent Technologies, Santa Clara, CA, USA). q-PCR was performed in a Stratagene Mx3005P thermal cycler using 40 ng of cDNA, Brilliant II SYBR Green qPCR Master Mix (Agilent Technologies), and 200–400 nM of primers targeting known Nlgn3 mouse and rat mRNA isoforms (Supplementary Table S1). Thermal cycling conditions included an initial activation step at 95 °C for 10 min and 40 cycles of denaturing at 95 °C, annealing at 60 °C, and amplification at 72 °C for 15 s. Amplification was checked for a single product by analyzing the melting curve, and the sizes of each product were confirmed by gel electrophoresis using GelRed Nucleic Acid Gel Stain (Biotium, Fremont, CA). The expression levels of Nlgn3 and cMyc were normalized to Rpl13a expression, using the delta–delta Cq method (2−ΔΔCq), as described.^96^

### shRNA experiments

Lentiviral constructs expressing shRNAs against mouse β-catenin (β-catenin-pLKO.1, #SHCLND-NM_007614) or control non-targeting shRNA (control-pLKO.1, #SHC002) were obtained from MISSION® (MISSION shRNA Library, Sigma-Aldrich). Lentiviral particles were produced in HEK293T cells co-transfected with the pLKO.1 construct, pCMV-dR8.91 (Delta 8.9) plasmid (containing gag, pol and rev genes) and pVSV.G plasmid (at a 3:2:1 ratio). Transfections were performed using Lipofectamine 2000 (Invitrogen, Thermo Fisher Scientific). Lentiviral particles were harvested 48 h post transfection, filtered through a cellulose acetate filter (0.45 µm) and concentrated by centrifugation (3,800 rpm at 4 °C for 30 min) with a 100 kDa Ultra15 Amicon filter (EMD Millipore, Billerica, MA, USA). Infections were carried out in HT22 cells plated in 60 mm diameter with 80–90 % confluence for 48 h using 100 µl concentrated virus.

### Western blot

Nlgn3, β-catenin and β-actin proteins were analyzed by western blot using the H-55 anti-Nlgn3 antibody (SC-50395; 1:1000; Santa Cruz Biotechnology, TX, USA), the monoclonal E-5 β- catenin antibody (SC-7963; 1:2000; Santa Cruz Biotechnology) or with the anti-β-actin antibodies (SC-47778; 1:5000; Santa Cruz Biotechnology). Membranes were washed (3×) and incubated with appropriate secondary antibodies conjugated to horseradish peroxidase for 1 h at room temperature, washed and incubated for 2 min with enhanced chemiluminescence solution (Pierce ECL, Thermo Scientific, IL, USA) and exposed for 1–3 min on Carestream Kodak BioMax films (Sigma-Aldrich). Secondary antibodies were: goat-anti rabbit IgG-HRP (SC-2004 for Nlgn3, 1:5000; Santa Cruz Biotechnology), goat-anti mouse IgG-HRP (SC-2055, for β- catenin and β-actin; 1:5000; Santa Cruz Biotechnology).

### Transcriptional activity of Nlgn3 promoter constructs

Nlgn3 promoter-luciferase fragments were generated by conventional PCR from genomic DNA extracted from either mouse HT22 hippocampal cells or Sprague-Dawley primary neurons using specific primers containing restriction sites (Supplementary Table S1) and subsequently inserted into the pGL3-Basic vector (Promega, Madison, WI, USA), as described.^94,95^ Activity of Nlgn3 promoters was measured in 80–90% confluent HEK293 cells seeded in 24 well culture plates. Cells were co-transfected using Lipofectamine 2000 (Invitrogen, Thermo Fisher Scientific) for 24 h with either pNL3 or pSUPERTOPFlash (STF) luciferase constructs,^97^ in the absence or presence of constitutively active β-catenin S33Y or the dominant negative ΔTCF4 constructs.^98^

The pRL-TK Renilla luciferase plasmid (1 ng) was used as an internal control. Firefly and Renilla luciferase activities were determined using the Dual-Luciferase Reporter Assay (Promega) in a Victor-3 multiplate reader (Perkin Elmer, Waltham, MA, USA). Promoter activity was normalized as the ratio between Firefly and Renilla luciferase units. Site-directed mutagenesis in TBE Sites II and III were generated using the pNL3Mm1.4 construct as background with M1 or M2 primers (Supplementary Table S1) with the QuickChange Site- Directed Mutagenesis kit (Stratagene, Agilent Technologies). All constructs were verified through direct sequencing (ABI-3130 Genetic Analyzer, Applied Biosystems, Foster City, CA, USA).

### Chromatin immunoprecipitation (ChIP) assays

ChIP studies were performed in HT22 neurons, as described.^94^ Briefly, 2.0 × 10^7^ cells were incubated with cell-conditioned medium containing Wnt3a (Wnt3a-CM; Wnt3a-CM 50% plus 50% of fresh HT22 incubation media), LiCl or CHIR 98014 (Sigma-Aldrich), as Wnt/β-catenin pharmacological agonists.^99,100^ Cells were cross-linked with 1% formaldehyde (Sigma-Aldrich) for 10 min at room temperature and then the reaction was stopped with 0.125 M glycine. Samples were sonicated with the M220 Focused-ultrasonicator (Covaris, Woburn, MA, USA) using milliTube caps (Covaris), following the high cell chromatin shearing protocol suggested by the manufacturer. In total 25 µg of chromatin was used in each ChIP assay. Endogenous β-catenin bound to TBE Sites within the mouse Nlgn3 promoter was immunoprecipitated with rabbit anti β-catenin antibody (SC-7199; 4 µg; Santa Cruz Biotechnology). Cross-linked chromatin fragments averaging 200–300 bp were assessed by q-PCR as described above. Antibody specificity was assayed with normal rabbit-IgG (12–370; 4 µg; EMD Millipore).

### Immunofluorescence and image processing

Control and Wnt3a-treated rat hippocampal neurons (14 DIV) cultured in poly-l-lysine (Sigma- Aldrich) coated coverslips (5.0 × 10^5^ cells/plate) were fixed using 4% formaldehyde-4% sucrose (pH: 7.0) solution for 20 min at room temperature. Cells were permeabilized with Triton X-100 0.2 % for 5 min and treated with blocking solution (PBS 1 × /BSA 2%, pH: 7,4) for 30 min. Primary antibodies against Nlgn3 (H-55; SC-50395; 1:100), PSD-95 (N-18; SC-6926; 1:100), and MAP2 (H-300; SC-20172; 1:100) (Santa Cruz Biotechnology, USA) were used and Alexa Fluor Antibodies (1:400; Molecular Probes, Eugene, OR, USA) for secondary staining. Samples were examined on a LSM780 confocal microscopy (Carl Zeiss, Jena, Germany). Images were deconvolved using the ImageJ/FIJI^101^ plugin DeconvolutionLab^102^ using theoretical point spread functions. Total Nlgn3 fluorescence intensity was measured as arbitrary units in all dendritic processes with clear signal of MAP2 in 15 µm segments that consistently covered an area between 40 and 200 µm^2^. To illustrate colocalization between Nlgn3 and PSD-95 proteins, three-dimensional isosurfaces of dendritic processes were created using Imaris v8.2 (Bitplane, Concord, MA, USA) and by applying the ImarisSurface tool (smoothness, 0.2 µm; quality level, 5) on the MAP2 fluorescent signal. Nlgn3 and PSD-95 clusters inside of dendritic processes were rendered using the ImarisSpots tool. Co-localization coefficients of Nlgn3 and PSD-95 (Pearson and Manders) were determined using the ImarisColoc tool in an average of four dendrites per neuron, which were selected based on brightness^103^ and length (~15 µm). Briefly, while Pearson’s correlation coefficient is an adimensional parameter that establishes whether colocalization exists independent of signal strength, thus avoiding the possibility of false positives, the Manders coefficient represents directly the percentage of colocalization between two signals.

### Statistical analysis

Data are presented as mean ± s.e.m. Each experiment was repeated three times with three replicates. All data processing and analysis were completed before unblinding of the analyzer. Sample size was chosen according to previous reports and our pre-experiments. A minimum number of three biological replicates were performed to ensure reproducible and robust changes. No cell samples or animals were excluded from the analysis. Data were analyzed with Prism v5 (GraphPad Software, La Jolla, CA, USA). For the comparison of more than two groups, we used one-way ANOVA test followed by Kruskal–Wallis *post hoc* test. To compare two groups, we performed a two-tailed Mann–Whitney test. Statistically significant p-values are shown as *P *<* 0.05, **P *<* 0.01 and ***P *<* 0.001.

## Results

### Enhancement of Nlgn3 expression via Wnt/β-catenin signaling in hippocampal cells

**Fig. 1:**
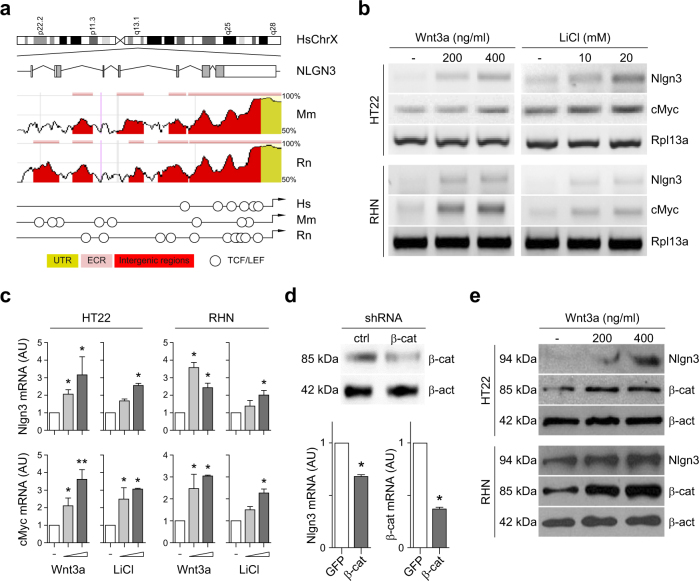
Wnt/β-catenin signaling activates Nlgn3 transcriptional program in hippocampal cells. **a** Top: Genomic context of human *NLGN3* in the long arm of chromosome X and schematic exon-intron boundaries of the gene. White and gray boxes: 5′ and 3′ UTR and exons, respectively. Middle: Conservation profile of the human *NLGN3* promoter sequence compared with similar genomic regions in *Mus musculus* and *Rattus norvegicus* (50–100%). Bottom: Schematic representation of potential TCF/LEF sites (TBE: CTTTG, circles) found in these species. ECR: Evolutionary conserved region. **b** Early expression levels of Nlgn3 and cMyc genes after 2 h treatment with increasing doses of either purified Wnt3a protein or LiCl in HT22 hippocampal cells or rat primary hippocampal neurons (RHN). Rpl13a was used as a reference gene. **c** Quantitative determination of Nlgn3 and cMyc mRNA levels after 2 h treatment with Wnt3a (200–400 ng/ml) protein or LiCl (10–20 mM) in HT22 hippocampal cells and RHNs. **d** TOP: protein levels of β-catenin after 48 h treatment with β-catenin-shRNA. shRNA of GFP was used as control and β-actin as a loading control. Bottom: expression levels of Nlgn3 and β- catenin. **e** Nlgn3 and β-catenin protein levels in HT22 cells and RHNs after 6 h treatment with 200 and 400 ng/ml of purified Wnt3a protein. β-actin was used as a loading control. In (**c**) and (**d**), data represent mean ± s.e.m., *P < 0.05, **P *<* 0.01, two-tailed Mann–Whitney test

According to genome wide data on β-catenin and TCF/LEF chromatin occupancy,^78,104–106^ most Wnt/β-catenin target genes have regulatory sequences within 2.5 kb clustering around transcriptional start sites (TSS). We searched for core TCF/LEF-binding elements (TBE: CTTTG) known to mediate β-catenin transcriptional activation of Wnt target genes, including a 3000 bp region upstream of the predicted transcriptional start site (TSS) in human, mouse and rat Nlgn3 gene sequences (Fig. 1a). We found multiple TBE sites in evolutionary conserved regions (ECR browser),^107^ suggesting a conserved role for the signaling cascade as a transcriptional regulator of Nlgn3 genes.

We have previously shown that Wnt/β-catenin target gene expression is rapidly induced (2–4 h) upon activation of the signaling cascade in different cell lines and primary cultures of hematopoietic cells.^94,95,108^ We therefore stimulated mouse HT22 hippocampal cells for 2 h with increasing concentrations of purified Wnt3a protein (200 and 400 ng/ml) or with LiCl (10 and 20 mM), which acts as a pharmacological inducer of Wnt/β-catenin signaling.^86,99^ We observed that both treatments increased Nlgn3 expression (Fig. 1b) and that the effect was paralleled by induction of the known Wnt/β-catenin target gene cMyc.^109^ The increase in Nlgn3 expression was consistently replicated in 14 DIV primary cultures of hippocampal neurons following short- term Wnt3a or LiCl treatments. Quantitative determination of mRNA levels in cells similarly treated confirmed that there was a significant increase in Nlgn3 and cMyc expression in response to Wnt/β-catenin activation (>2.5 and >3-fold induction, respectively; *P < 0.05; *n* = 3) (Fig. 1c) and that the transcriptional effect was clearly observed after 4 h treatment. Conversely, infection of HT22 cells with shRNA against mouse β-catenin for 48 h significantly decreased endogenous Nlgn3 expression (up to 35%; Fig. 1d). In addition, augmented protein levels of β-catenin and Nlgn3 were readily observed in HT22 cells and hippocampal neurons after 6 h Wnt3a treatment (200 and 400 ng/ml; Fig. 1e), at a time when most β-catenin is found within the nucleus of these cells (Supplementary Figure S1). We concluded that Wnt/β-catenin signaling is involved in the transcriptional program that controls Nlgn3 expression.

### Contribution of TCF/LEF binding elements in mammalian Nlgn3 promoter activity

**Fig. 2:**
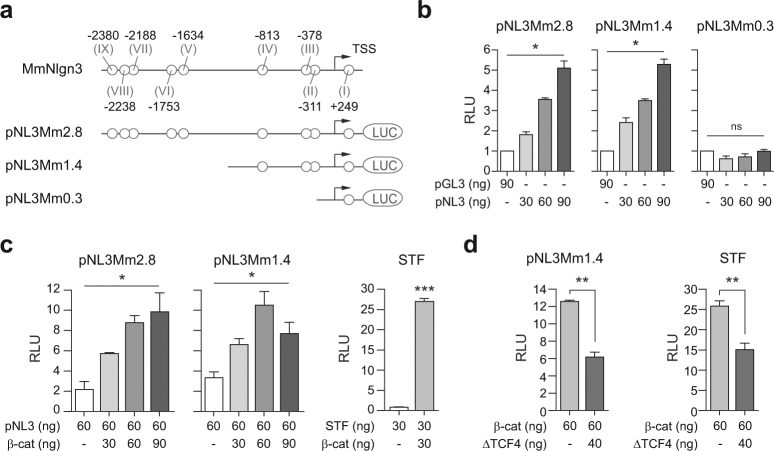
Contribution of Wnt/β-catenin responsive TBE sites to Nlgn3 promoter activity. **a** Genomic context of mouse Nlgn3 promoter and different Nlgn3 chimeric promoter constructs (pNL3Mm). Circles indicate potential TBE consensus sequences in the predicted regulatory sequence of Nlgn3 gene. Distances are in reference to the transcription start site (TSS). **b** Transient transfection of increasing doses (30, 60, and 90 ng) of Nlgn3 chimeric promoter constructs pNL3Mm2.8, pNL3Mm1.4, and pNL3Mm0.3 in HEK293 cells for 24 h. **c** Transient co-transfection of pNL3Mm2.8 kb or pNL3Mm1.4 with increasing doses of constitutively active β-catenin (S33Y) for 24 h. **d** Effects of dominant negative delta-TCF4 (ΔTCF4) on β-catenin induced pNL3Mm1.4 promoter activity. In (**c**, **d**) SuperTOPFLASH (STF) activity was measured as a positive control for Wnt/β-catenin pathway activation. RLU: relative luciferase units. In (**b**, **c**) Data represent mean ± s.e.m., *P < 0.05, one-way ANOVA and Kruskal–Wallis post hoc test. In (**d**) Data represent mean ± s.e.m., **P *<* 0.01, two-tailed Mann–Whitney test. ns not significant

To investigate the contribution of Wnt/β-catenin responsive TBE sites on the transcriptional activity of the mouse Nlgn3 promoter, we initially cloned a 2849 bp genomic segment upstream of the luciferase gene in the pGL3-basic vector (pNL3Mm2.8) and assayed its activity through transient transfections in HEK293 cells. This promoter region included 2448 bp upstream of the TSS (Nlgn3-001 transcript, ENSMUST00000065858 used as reference), 87 bp of the unstranslated exon 1 and 314 bp of intron 1, contained nine putative TBE sites (Fig. 2a) and displayed significant basal transcriptional activity (Fig. 2b), indicating that the pNL3Mm2.8 reporter maintains necessary elements to support Nlgn3 gene expression. As suspected, a significant enhancement in pNL3Mm2.8 reporter activity occurred when HEK293 cells were co-transfected for 24 h with increasing concentrations of a construct coding for a constitutively active β-catenin (S33Y)^98^ protein (ca. 5 fold promoter activity; *P* < 0.05, *n* = 3; Fig. 2c).

To identify the minimal promoter with maximal response to Wnt/β-catenin signaling, we serially-deleted potential TBE sites, using the pNL3Mm2.8 construct as background. We observed that the activity of pNL3Mm1.4 (1,362 bp, −961/+401), containing TBE sites I–IV and basal elements, was significantly augmented in the presence of constitutively active β-catenin (S33Y) co-transfected for 24 h (>3-fold; *p* < 0.05; Fig. 2c). In addition, we observed that a third 244 bp construct (pNL3Mm0.3) containing only intron 1 and TBE site I (+157/+401) did not support basal reporter transcription (Fig. 2b) and thus it was discarded for subsequent experiments. Next, the transcriptional activity pNL3Mm1.4 was explored in loss-of-function assays with a dominant-negative TCF4 (ΔTCF4) construct, which codes for a transcription factor lacking 30 residues from its amino-terminus and that is unable to bind β-catenin.^98^ We found that the enhancement induced by β-catenin on pNL3Mm1.4 promoter activity was significantly antagonized ( > 50%) by co-expressing ΔTCF4 for 24 h (Fig. 2d), corroborating that β-catenin- mediated transcriptional activation involves association with TCF/LEF family members. Finally, these gain and loss-of-function experiments were consistently replicated using four additional rat Nlgn3 promoter constructs (pNL3Rn2.8: 2098 bp, −1786/+312; pNL3Rn1.4: 1377 bp, −1065/+312, pNL3Rn1.1: 1116 bp, −1786/−671; and pNL3Rn0.6: 566 bp, −2098/−1221; Supplementary Figure S2), indicating that pNL3-1.4 represents an evolutionary conserved promoter region, which exhibits basal activity and a significant induction in response to Wnt/β-catenin modulation.

### Novel and functional TBE site in the promoter of the Nlgn3 gene

**Fig. 3:**
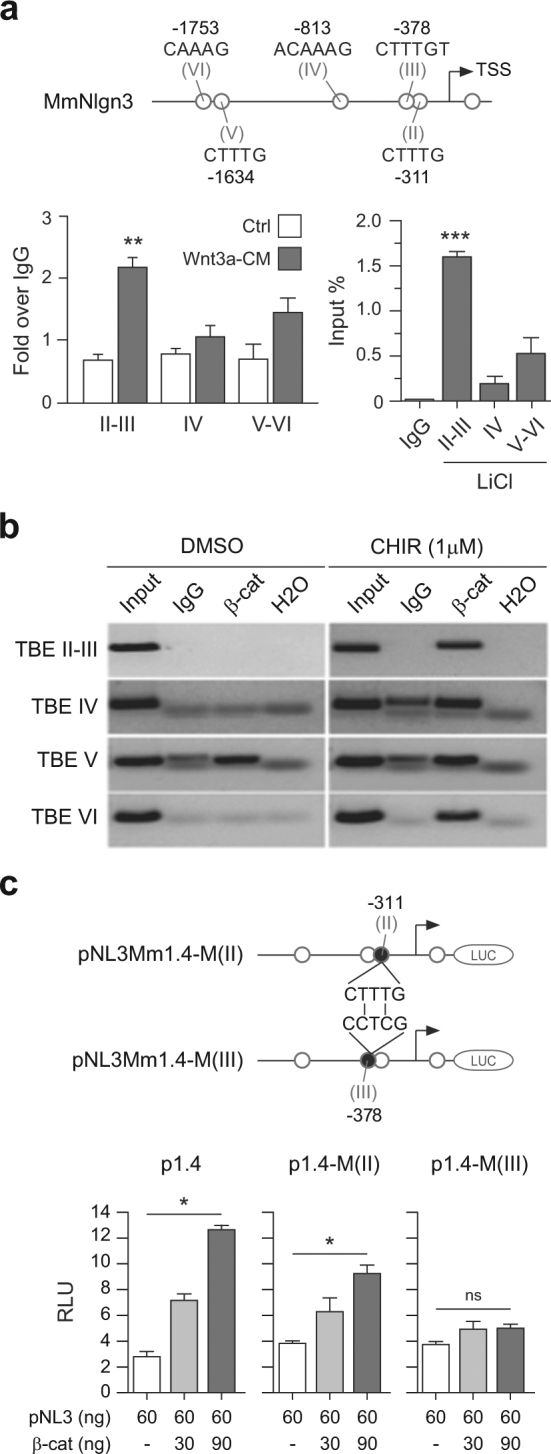
Functional β-catenin responsive element in the Nlgn3 promoter. **a** Top panel: Representation of the 1.4 kb mouse Nlng3 promoter construct depicting the five TBE sites analyzed by ChIP assays. Bottom panel: Endogenous β-catenin binding to TBE Sites in the mouse Nlgn3 promoter examined in HT22 cells after treatment with either Wnt3a-CM for 4 h (left) or 20 mM LiCl for 24 h (right). Data represent mean ± s.e.m., **P *<* 0.01, ***P *<* 0.001, two-tailed Mann–Whitney test. **b** TBE sites (II–VI) were similarly examined by ChIP assays in HT22 cells under control conditions or treated with 1 µM CHIR 98014 for 4 h. In (**a** and **b**) immunoglobulin G (IgG) was used as a control. **c** Top panel: Representation of changes introduced by site-directed mutagenesis in TBE Sites II and III in the context of the 1.4 kb mouse Nlng3 promoter construct. TBE consensus sequence: CTTTG; Mutated TBE sequence: CCTCG. Bottom panel: Transient transfection of increasing doses of mutant plasmids in the presence of constitutively active β-catenin (S33Y) in HEK293 cells for 24 h. RLU: relative luciferase units. Data represent mean ± s.e.m., *P < 0.05, one-way ANOVA and Kruskal–Wallis post hoc test. ns not significant

The binding of endogenous β-catenin to conserved TBE sites on the Nlgn3 promoter was analyzed through chromatin immunoprecipitation (ChIP) assays in HT22 cells. We observed that a significant binding of β-catenin to TBE sites II and III within the mouse Nlgn3 promoter was induced after treatment with either cell-conditioned media containing Wnt3a (Wnt3a-CM, see Methods) for 4 h or 20 mM LiCl for 24 h (rapid or persistent response, respectively) (Fig. 2a). In addition, direct binding of β-catenin to TBE sites II–III was similarly observed in HT22 cells incubated for 4 h with CHIR 98014 (Fig. 2b), which acts as a specific inhibitor of GSK3β activity and thus enhances nuclear and cytosolic levels of β-catenin.^100^ Finally, since these experiments did not resolve whether β-catenin binds directly to Nlgn3 TBE Site II and/or TBE Site III (mainly due to their close spatial proximity), we introduced through site-directed mutagenesis two-nucleotide changes in each TBE sequence, using pNL3Mm1.4 as the template (Fig. 3c). Remarkably, transient transfection of these constructs in HEK293 cells for 24 h revealed that mutations affecting only the consensus sequence of TBE site III (pNL3Mm1.4-MIII) completely abrogated β-catenin activation of the pNL3Mm1.4 reporter construct. From these experiments, we conclude that TBE site III (−378/−382) is a functional Wnt/β-catenin transcriptional element within the Nlgn3 promoter.

### Wnt/β-catenin signaling enhances Nlgn3 clustering

**Fig. 4:**
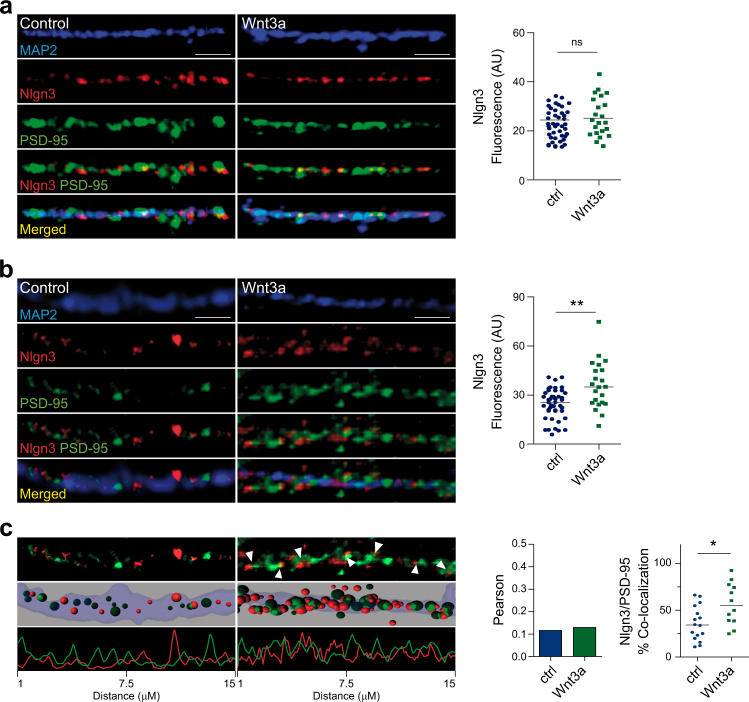
Wnt/β-catenin signaling enhances Nlgn3 levels and synaptic clustering in hippocampal neurons. **a** Left panel: Representative confocal images of dendritic processes showing MAP2 (blue), Nlgn3 (red), and PSD-95 (green) immunofluorescence signals in primary hippocampal neurons under control conditions or treated with 400 ng/ml of purified Wnt3a protein for 2 h. White bar represents 2.5 µm. Right panel: Total Nlgn3 intensity fluorescence. **b** Same as in (**a**) but after 24 h Wnt3a treatment. **c** Left panel: Three-dimensional isosurface rendering of dendritic processes (smoothness, 0.2 µm; quality level, 5) and florescence intensity of Nlgn3 and PSD-95 clusters. Right panel: Colocalization coefficients for Nlgn3–PSD-95 interaction in control versus Wnt3a-treated neurons (Pearson and Manders, respectively). Each figure corresponds to three independents experiments (**a** ctrl *n* = 44 and Wnt3a *n* = 21; **b** ctrl *n* = 44 and Wnt3a *n* = 22; **c** ctrl *n* = 16 and Wnt3a *n* = 12). Data represent mean, *P < 0.05, **P < 0.01, two- tailed Mann–Whitney test. ns not significant

Nlgn3 promotes synapse formation and localizes at excitatory or inhibitory synapses, where it interacts with postsynaptic proteins PSD-95 or gephyrin, respectively.^29,30,32^ Given that we reported that purified Wnt3a modulated intracellular calcium and enhanced excitatory neurotransmission in hippocampal neurons,^84^ we hypothesized that the signaling cascade may enhance synaptic function by increasing Nlgn3 levels in dendritic processes, where it would interact with PSD-95 in the postsynaptic terminal. Therefore, we incubated 14 DIV rat hippocampal neurons in the absence or presence of purified Wnt3a (400 ng/ml) for 2 or 24 h and performed immunofluorescence analyses to examine whether activation of the signaling cascade affects the differential recruitment of Nlgn3 to dendritic processes. First, Nlgn3 was distributed along dendrites in a punctate manner, in agreement to previous reports,^32^ where it regularly colocalized with PSD-95 protein (Fig. 4a). Notably, after 24 h Wnt3a incubation we observed that the average Nlgn3 intensity fluorescence was significantly increased in dendrites compared to control primary neurons (Fig. 4b). We were not able to detect differences in Nlgn3 fluorescence after short-term Wnt3a treatment (2 h). Second, we examined the effect of the Wnt3a ligand in Nlgn3 and PSD-95 clustering by measuring the intensity and surface area of individual clusters in dendritic processes. We observed that both Nlgn3 and PSD-95 cluster intensity and surface area increased after 24 h Wnt3a treatment (Supplementary Figure S3a), indicating that these excitatory synaptic proteins are differentially recruited to potential synaptic sites. We further measured the area covered by these proteins along dendrites and observed that Nlgn3 surface area, but not PSD-95 surface area, was significantly increased after 24 h Wnt3a treatment (Supplementary Figure S3b). Finally, three-dimensional volume rendering of Nlgn3 and PSD-95 clusters further revealed a significant overlap of Nlgn3 and PSD-95 signals (Fig. 4c), and confirmed that Wnt3a signaling induces Nlgn3 clustering and colocalization with PSD-95 protein, likely participating in excitatory postsynaptic assembly.

## DISCUSSION

An excess of synapses in frontal, temporal and parietal lobes has been found in children with ASD compared to age-matched controls^110,111^ and changes in synaptic structure are detected in multiple mouse models of ASD.^112^ In this context, due to its participation in synaptic structure and maturation since early development, postsynaptic cell-adhesion molecules such as Nlgns and associated partners are major candidate genes to understand ASD. Nevertheless, very little is known regarding the transcriptional program that controls the expression of these molecules. Here we have shown that Wnt/β-catenin signaling induces a dose-dependent enhancement of Nlgn3 mRNA and protein levels, which is observed soon after induction of the signaling cascade and within the time frame observed for several other Wnt/β-catenin target genes whose promoters contain TBE sites.^77,113^ We detected that endogenous β-catenin was predominantly associated with the Nlgn3 promoter region spanning TBE site III (−378/−382) under control conditions, and that such interaction was clearly enhanced when cells were incubated in presence of signaling agonists. While our results represent the first functional characterization of the Nlgn3 promoter, we note that Nlgn1 and Nlgn2 have already been predicted by bioinformatics approaches as potential β-catenin target genes.^78^ Accordingly, activation of the signaling cascade by the chemical compound curcumin has been shown to effectively enhance Nlgn1 expression in endogenous neural stem cells.^114^ Interestingly, curcumin has shown anti- inflammatory and antioxidant properties and is being considered as a therapeutic option in cancer and various prevalent neurological disorders, including ASD.^115–117^

It is widely accepted that Wnt/β-catenin signaling is essential for brain development and function and is increasingly recognized that the cascade has a central role in ASD neurodevelopmental pathology. Indeed, Wnt/β-catenin signaling is involved in neurogenesis,^118^ axonal remodeling,^119^ patterning and maturation of functional synapses^120–122^ and excitatory neurotransmission.^82–85^ Likewise, increased β-catenin levels are critical for synaptic structure and dendritic arborization.^123–125^ In this regard, increased Nlgn3 intensity and Nlgn3 co-localization with PSD-95 after 24 h Wnt3a treatment supports the idea that the signaling cascade is involved in excitatory synaptogenesis through recruiting and clustering of proteins in dendritic processes. For instance, while large clusters of PSD-95 are indicative of spine stabilization^126^ it has been observed that similar treatment of hippocampal neurons with canonical Wnt7a, consistently increased PSD-95 puncta in a time-dependent manner and in a calcium/calmodulin-dependent protein kinase II (CAMKII) dependent mechanism.^122^ Interestingly, CAMKII activation is dependent of calcium entrance into the synaptic terminal and our group has already shown that Wnt3a increases transient calcium currents.^84^ Altogether, these data support a regulatory role for this kinase in ASD and other neurodevelopmental diseases.^127,128^

Cortical neuronal activity exerts mitogenic effects on neural and oligodendroglial precursor cells^129^ and recent experiments have shown that this neuronal activity involves the shedding of the Nlgn3-extracellular-domain from the postsynaptic surface.^130^ It would be interesting to examine whether Nlgn3 expression and clustering promoted by the Wnt/β-catenin signaling cascade leads to Nlgn3 shedding and how this process relates to neuroinflammation and defects in excitatory/inhibitory neurotransmission.^131,132^

## Electronic supplementary material

Supplementary Figure S1

Supplementary Figure S2

Supplementary Figure S3

Supplementary Table S1
