# Supplementary figures and images for: Wnt/β-catenin signaling stimulates the expression and synaptic clustering of the autism-associated Neuroligin 3 gene
Source: Transl Psychiatry. 2018 Mar 5;8:45. doi: 10.1038/s41398-018-0093-y (PMC5835496; doi:10.1038/s41398-018-0093-y)

Supplementary Figure S1

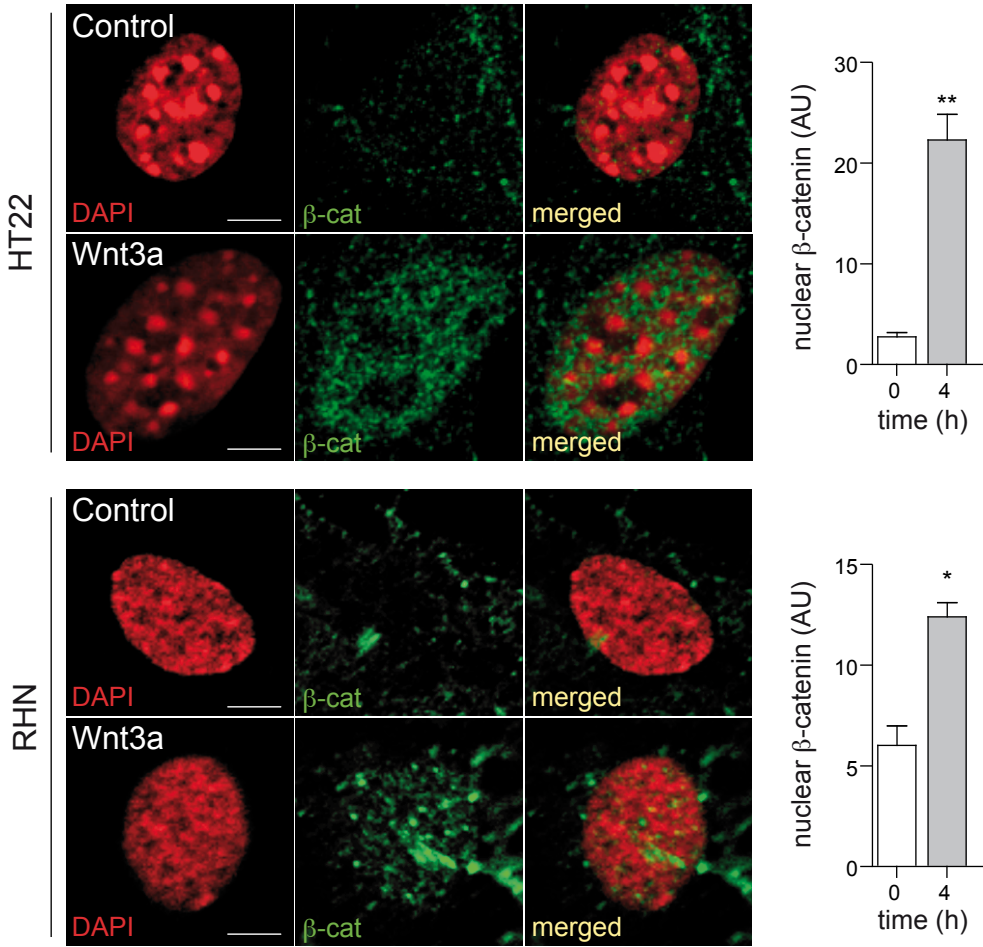

Supplement: Supplementary file 1 — Supplementary Figure S1 [file 41398_2018_93_MOESM1_ESM.pdf]

Supplementary Figure S2

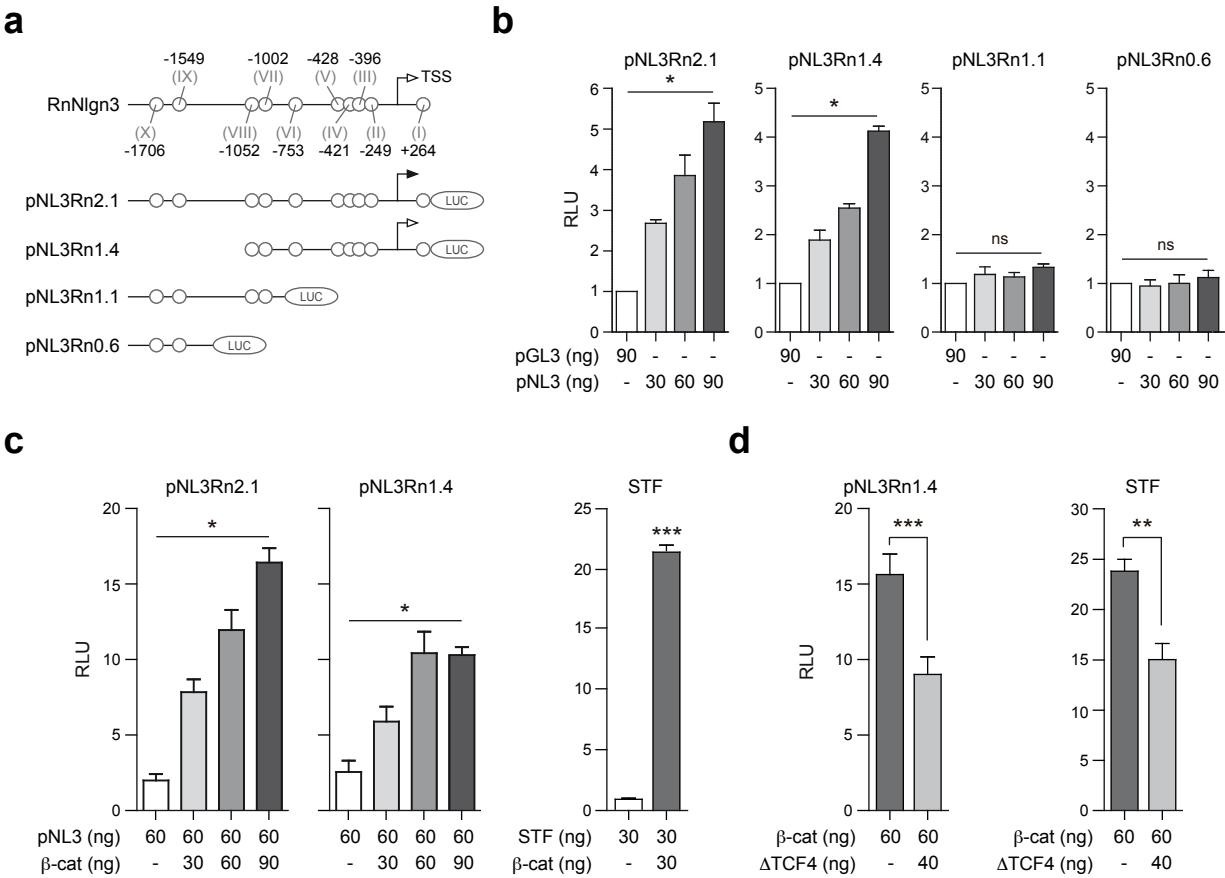

Supplement: Supplementary file 2 — Supplementary Figure S2 [file 41398_2018_93_MOESM2_ESM.pdf]

## Supplementary Figure S3

**a**

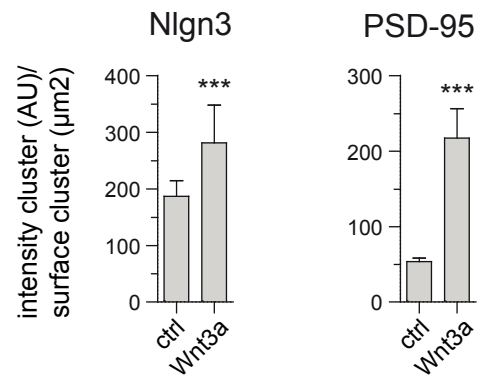

**b**

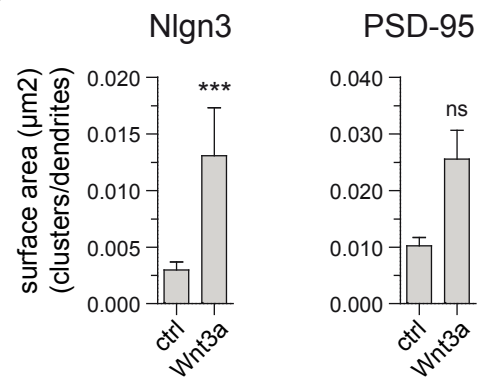

Supplement: Supplementary file 3 — Supplementary Figure S3 [file 41398_2018_93_MOESM3_ESM.pdf]
