# Supplementary material for: Wnt/β-catenin signaling stimulates the expression and synaptic clustering of the autism-associated Neuroligin 3 gene
Source: Transl Psychiatry. 2018 Mar 5;8:45. doi: 10.1038/s41398-018-0093-y (PMC5835496; doi:10.1038/s41398-018-0093-y)
Supplement: Supplementary file 4 — Supplementary Table S1 [file 41398_2018_93_MOESM4_ESM.pdf]

Supplementary Table S1. Primers used in our study.

assay	primer	sequence
**qPCR**	Nlgn3Mm-FW	CATGGAAGGAACAGGCAACA
	Nlgn3Mm-RV	ACGGGGATCTCCTCCAAAGA
	Rpl13aMm_FW	CACTCTGGAGGAGAAACGGAAGG
	Rpl13aMm_RV	GCAGGCATGAGGCAAACAGTC
	CmycMm_FW	CTGCTGTCCTCCGAGTCCT
	CmycMm_RV	GCCTCTTCTCCACAGACACC
	Ctnnb1Mm_FW	CAGCGCTGGTGAAAATGCTT
	Ctnnb1Mm_RV	CTGCAGTCCACCAGCTAGGC
	Nlgn3Rn-FW	CCCTATGTTTTTGGTGTCCCTA
	Nlgn3Rn-RV	CCTTGGTGTGAATGAACTTGG
	Rpl13aRn_FW	ACAAGAAAAAGCGGATGGTG
	Rpl13aRn_RV	TTCCGGTAATGGATCTTTGC
	CmycRn_FW	TCTCTTCTTCCTCGGACTCG
	CmycRn_RV	CACCACATCAATTTCTTCCTCA
**Cloning**	pNL3Mm-XhoI-FW	GGTGCTAAGGAGAGGTGATGCT
	pNL3Mm-BglII-RV1	GAAGATCTTCTGGGGGCTTAGAGGAA
	pNL3Mm-BglII-RV2	GAAGATCTTCTTGGCAATGTGCAATC
	pNL3Rn-FW	TGAAACATTGAACTGTTGGGTTTTG
	pNL3Rn-RV	GATGATAGGGATGCAGGATTTAGGG
**ChIP**	Nlgn3_Fw_TBE2-3	GTGATTGCACCTTGGGTGGT
	Nlgn3_Rv_TBE2-3	ACACACACACCCACCACGTC
	Nlgn3_Fw_TBE4	CCCTATCACTGGGAGGTGTCA
	Nlgn3_Rv_TBE4	TGCTTCAGTTGTGCTGGCTTT
	Nlgn3_Fw_TBE5	TGTGAGAGTACCGGGGAAAGG
	Nlgn3_Rv_TBE5	CTTGGAGCTCATTCGGGTTG
	Nlgn3_Fw_TBE6	GAAACATTGAACTGTTGGGTTTTG
	Nlgn3_Rv_TBE6	GAGGAGGGAGACGATGCAGA
